# Recognizing pediatric febrile myoclonus: a video-documented case report and review of the current literature

**DOI:** 10.3389/fped.2025.1653744

**Published:** 2025-08-26

**Authors:** M. Minerva, L. Perilli, A. Francioni, F. Lotti, M. R. Curcio, S. Grosso

**Affiliations:** Clinical Pediatrics, Department of Molecular Medicine and Development, Azienda Ospedaliero-Universitaria Senese, University of Siena, Siena, Italy

**Keywords:** febrile myoclonus, movement disorder, EEG, infant—age, pediatric neurology and psychiatry

## Abstract

Febrile myoclonus (FM) is a benign condition characterized by the sudden onset of generalized, multifocal, or segmental jerks during fever, without signs of central nervous system infections, resolving as the temperature elevation subsides. This condition is poorly described in literature, and its incidence is not well identified. We present the case of a 1-year-old child who developed myoclonic jerks during a febrile episode, diagnosed with FM. The child exhibited a normal neurological examination and neuromotor development appropriate for age. The electroencephalogram (EEG) showed no epileptiform discharges during the events, and past medical or familiar history was negative for epilepsy or any other neurological condition. We conducted a narrative review of the current literature to improve understanding of this benign clinical manifestation. Additionally, we included audio and video material to aid physicians in recognizing the condition, avoiding unnecessary diagnostic procedures and overtreatment with antiseizure medications. Our goal is to increase awareness of this rare phenomenon and expand its phenotypical spectrum.

## 1 Introduction

Myoclonus is defined as a brief, sudden contraction or interruption of ongoing muscular activity, caused by abnormal electrical discharges in the central or peripheral nervous system (1). Its classification, based on the underlying physiology and the diversity of causes, guides therapeutic decisions (2). It can occur at any age, either as an isolated phenomenon or associated with other neurological diseases (3).

Myoclonus can be further classified by distribution (focal, multifocal and generalized), by provoking factors (spontaneous, reflex) or by site of generation (cortical, subcortical, spinal or peripheral) (4). A detailed clinical assessment is critical to determine the site of onset, patterns, sensitivity to stimuli, and associated neurological signs. This is supported by diagnostic tools such as electromyography (EMG), EEG-EMG polygraphy, jerk-locked back averaging, evoked potentials, and long-latency reflex (C-reflex) (1).

In children, myoclonus is often transient and underrecognized, particularly when associated with fever. Despite being described in literature (5–12), FM remains frequently misinterpreted, leading to unnecessary investigations and treatment. Prompt clinical recognition is therefore crucial to avoid misdiagnosis. This report presents a case of febrile generalized myoclonus in a previously healthy 1-year-old child, accompanied by a narrative review to support clinicians in identifying this rare condition, alongside video and audio material of the events.

## 2 Materials and methods

An extensive literature search was conducted in PubMed in January 2025 to identify English-language case reports of pediatric FM. The search strategy employed keyword combinations using the terms: (febrile myoclonus) AND (“pediatric” OR “child” OR “infant” OR “toddler”), yielding a total of 115 articles. Inclusion criteria were case reports describing episodes of myoclonus occurring in pediatric patients during febrile illness. Exclusion criteria encompassed articles not written in English and those not relevant to the topic. After the screening, 9 articles were deemed suitable and included in the final analysis.

## 3 Case description

We present the case of a male patient, born at 40 + 1 weeks of gestational age via operative vaginal delivery. The APGAR scores were 9 at 1 min and 10 at 5 min. Pregnancy history and fetal ultrasound follow-up were unremarkable. Auxological parameters were within normal limits. Neurodevelopmental milestones were appropriately achieved according to the expected timeline. Past medical history included bronchiolitis requiring ventilatory support in the neonatal intensive care unit. Family and personal medical histories were negative for epilepsy or any other significant neurological disorder.

At 1 year of age the patient presented with paroxysmal episodes occurring during febrile peaks (39°C), predominantly when falling asleep. Upon presentation, the patient exhibited rhinitis and cough consistent with an upper respiratory tract infection. Blood tests and blood cultures were within normal limits, pharyngeal swab tested positive for Bocavirus. Neurological examination was otherwise unremarkable, and therefore lumbar puncture was not performed. These manifestations were characterized by rapid, massive muscle contractions involving all four limbs, sporadically associated with eye-rolling, followed by crying. During these episodes, altered mental status was observed. The episodes lasted a few seconds, occurred in clusters, resolving concomitantly with antipyretic administration and subsequent defervescence.

**Figure 1:**
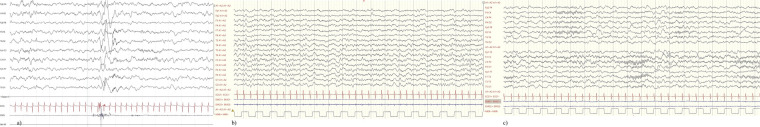
**(a)** Brief muscle jerks of the upper limbs resulting in a movement artifact without electric alterations; **(b)** awake EEG showing normal background activity and regional differentiation; **(c)** sleep EEG showing normal NREM sleep elements.

Given the high frequency of episodes initially interpreted as seizures, and the close succession of febrile peaks during recovery, a continuous infusion of midazolam at subanesthetic dose was initiated (0.04 mg/kg/h) but promptly discontinued due to sedation and suspicion of FM. After washout of the benzodiazepine, EEG recording during fever revealed a brief episode of muscle contractions not associated with epileptiform discharges (Figure 1a). The clinical features, combined with EEG findings, supported a diagnosis of a paroxysmal non-epileptic manifestation, specifically febrile generalized myoclonus.

Since admission, the patient experienced four episodes with similar characteristics. Follow-up EEG demonstrated normal background activity, regional differentiation, and normal sleep-phase architecture, without epileptiform activity (Figure 1). After resolution of the upper tract infection and defervescence no more paroxysmal episodes occurred. Audio and video recordings, along with EEG tracings collected during recovery, are included in this manuscript with informed consent from the patient's family (Supplementary Video S1). At last follow-up in July 2025 (1 year and 5 months of age) the child experienced additional myoclonus episodes isolated during febrile events, negative EEG and no neurological sequelae, including signs of neurodevelopmental stagnation or regression (Table 1).

**Table 1:** Chronological overview of symptom onset, diagnostic considerations, therapeutic interventions, and follow-up.

Day/Timepoint	Event	Details
Day 1	Onset of fever and febrile myoclonus	First episode of febrile myoclonus; continuous IV midazolam initiated (0.04 mg/kg/h)
Day 2	EEG and treatment adjustment	Midazolam promptly discontinued; EEG recorded febrile myoclonus without epileptiform activity
Day 3	Resolution of symptoms	Fever and febrile myoclonus resolved
Day 4	EEG	Normal
1 month follow-up	Follow-up child neurology consultation	New febrile episode with concomitant myoclonus managed at home
5 months follow-up	Follow-up child neurology consultation	New myoclonus clusters during febrile peak managed at home Normal neuromotor development, normal EEG

## 4 Discussion

### 4.1 Clinical characteristics and classification

Myoclonus is a clinical phenomenon that can be classified as physiologic, essential, epileptic, symptomatic, or psychogenic, originating from various levels of the central nervous system (13). Febrile neurological events in children include seizures, delirium, and less frequently FM, a rare, benign and underdiagnosed condition. FM typically affects children from 6 months to 6 years old, similarly to febrile seizures, and resolves with defervescence. Most cases show normal neurological examination, neurodevelopment and EEG findings. FM clusters can last from a few seconds up to 30 min, occasionally extending up to 24 h (5, 14).

### 4.2 Age range and presentation

Although most cases described in the literature occur within the commonly reported age range, various report support a broader spectrum. For instance, Onoe et al. reported on a child aged 11 years and 7 months (5), while a survey by Rajakumar et al. included a case of a 10-year-old child (6). FM typically appears within the first 3 days of fever, mainly at body temperatures above 38.5°C. This latter finding was consistent across all reported cases, including our patient. In rare instances, post-infectious afebrile myoclonus has been documented, for example after enterovirus or influenza infections (7). As noted, most patients exhibit unremarkable neurological examinations and age-appropriate neurodevelopment (5, 8–11). Exceptionally, Delucchi reported a child with hypotonia, as well as walking and speech disorders, resulting from a severe perinatal insult. Despite EEG recording showed multifocal sharp waves, the myoclonic events were not associated with generalized discharges, supporting a diagnosis of FM.

### 4.3 EEG findings

EEG findings are often normal or show transient abnormalities. In the cohort reported by Onoe et al., four patients exhibited epileptiform patterns such as asymmetry of the posterior dominant rhythm, diffuse spike-wave paroxysms, or focal occipital slowing. In most cases, these abnormalities resolved within days, concomitant with FM resolution (5).

### 4.4 Pathophysiology

The pathophysiology of FM is not well understood, but some authors hypothesize that emotional symptoms, often seen with myoclonus, might arise from dysfunction in the hypothalamus and basal ganglia, causing both myoclonic jerks and mood changes. Although specific viral strains are suspected to contribute to FM pathogenesis, younger children, being more susceptible to severe infections, might exhibit exaggerated CNS responses, possibly explaining the transient and benign nature of FM and its typical age range (12).

### 4.5 Differential diagnosis

Febrile and post-infectious myoclonus in children may arise from various conditions. In the differential diagnosis, drug- or toxin-induced myoclonus should always be considered, whether associated with fever or occurring in the absence of infection (15). Autoimmune encephalitis can present with fever, myoclonus, seizures with a EEG correlate, and behavioral changes, particularly in pediatric patients (16). Post-infectious acute cerebellar ataxia with myoclonus may follow viral illnesses, manifesting with ataxia, myoclonus, and sometimes additional cerebellar signs; most cases are benign and self-limited (17). Opsoclonus-myoclonus syndrome, a rare neuroimmunologic disorder, is typically parainfectious or paraneoplastic (e.g., neuroblastoma) and presents with myoclonus, ataxia, and behavioral changes (18). Finally, Sydenham chorea remains the most common cause of acute chorea in children, usually in association with acute rheumatic fever or following Group A Streptococcus infection, and is characterized by purposeless, involuntary movements, often accompanied by emotional lability and muscle weakness (19).

Although all these diagnoses are compatible with a history of infectious-related pediatric movement disorder, the anamnestic, clinical, and instrumental data did not support these diagnostic hypotheses in our patient.

## 5 Limitations

This report is limited by its anecdotal nature, short follow-up (5 months), and lack of jerk-locked EEG or long-term EEG/video EEG. Although the clinical features and EEG findings suggest a benign course, longer follow-up is necessary to exclude progression to epileptic disorders. The literature lacks standardized diagnostic criteria for FM, with diagnosis primarily relying on clinical expertise and phenotypic observation.

## 6 Conclusions

FM is a rare, benign, and likely underdiagnosed paroxysmal event in children. Differentiating it from epileptic seizures is essential to avoid unnecessary treatments, as misdiagnosis may lead to prolonged use of antiseizure medications with potential adverse effects and challenges in discontinuation. Although follow-up is advisable, most cases resolve without sequelae. By reporting this case and reviewing the available evidence, we aim to raise awareness among clinicians and highlight the need for further research into the incidence and underlying mechanisms of FM.

## Data Availability

The datasets presented in this article are not readily available because of ethical and privacy restrictions. Requests to access the datasets should be directed to the corresponding author.

## Supplementary material

Supplementary Video S1 Two episodes of paroxysmal myoclonic jerks involving all four limbs, associated with eye rolling and followed by crying, occurred during a rise in temperature and while falling asleep. The adult's hand movements in the second half of the video are used to assess the child's state of consciousness and responsiveness, in order to evaluate their reaction to such a stimulus.
